# An engineered three-dimensional gastric tumor culture model for evaluating the antitumor activity of immune cells *in vitro*

**DOI:** 10.3892/ol.2012.1021

**Published:** 2012-11-09

**Authors:** PEIMING SUN, YINGXIN XU, XIAOHUI DU, NING NING, HUIWEI SUN, WENTAO LIANG, RONG LI

**Affiliations:** Institute of General Surgery, Chinese PLA General Hospital, Haidian, Beijing 100853, P.R. China

**Keywords:** immune cells, antitumor activity, three-dimensional culture, tumor model, gastric cancer

## Abstract

Monolayer tumor culture models have been used for evaluating the antitumor activity of immune cells *in vitro*. However, their value in this research is limited. We used human gastric cancer cells (BGC823) and collagen hydrogel as a matrix to establish an engineered three-dimensional (3-D) tumor culture model *in vitro*. Tumor cells grew in 3-D culture and formed spheroids in the collagen matrix. Evaluation of the antitumor activity of cytokine-induced killer (CIK) cells revealed that, compared with the 2-D cell culture models, CIK cells migrated towards the tumor cells and destroyed the spheroids and tumor cells in the engineered 3-D tumor culture model. The cytotoxicity of CIK cells against the tumor cells in the engineered 3-D tumor culture model was lower than that in 2-D tumor culture models at 12–36 h post-interaction, but there was no significant difference in the cytotoxicity at later time points. Further analysis indicated that dendritic cell-activated CIK cells had a significantly higher level of cytotoxicity against tumor cells, compared with CIK and anti-CEA/CD3-treated CIK cells, in the engineered 3-D tumor culture model. Our data suggest that the engineered 3-D gastric tumor culture model may better mimic the interaction of immune cells with tumor cells *in vivo* than the 2-D tumor culture models, and may be used for evaluating the antitumor activity of immune cells *in vitro*.

## Introduction

Traditionally, the biological behavior of solid tumors and their responses to immune cells *in vitro* are examined using stabilized tumor cell lines grown in a two-dimensional (2-D) monolayer culture (1). Although this strategy has provided valuable information with regard to the cytotoxicity of immune cells to tumor cells, it is not sufficient to mimic the antitumor activity of immune cells *in vivo*. Solid tumor cells grown in three-dimensions (3-D) *in vivo* support each other and also interact with the extracellular matrix (ECM). The behavior of a single tumor cell is regulated by its interactions with its immediate neighbors and the ECM (2). Similarly, the antitumor activity of immune cells may be affected by the 3-D spatial array and ECM (3,4). In this aspect, the *in vitro* 2-D tumor cell culture model has limited value in the evaluation of the clinical efficacy of the cytotoxicity of immune cells *in vivo*. Therefore, it is necessary to establish an *in vitro* tumor model that accurately reflects the *in vivo* immune cell-tumor cell interactions. Such *in vitro* models may be used for successful screening of tumor-reactive immune cells prior to testing their *in vivo* antitumor immunity in animal models, as well as for the treatment of patients.

Tissue engineering practices have suggested that the 3-D culture of cell models *in vitro* is better than the planar cultures of cell lines, particularly in mimicking the *in vivo* cell-cell and cell-matrix interactions (5). Furthermore, it has been reported that the cellular microenvironment, cell-cell and cell-matrix interactions within 3-D tissues greatly influence cellular functions, including the adhesion, motility, invasiveness and metastasis of cancers (6). Thus, 3-D culture may be an important strategy for the evaluation of the behavior of tumor cells and the efficacy of immunotherapeutic intervention. There are various methods available for the establishment of 3-D tumor cultures *in vitro*, including the spinner flask method (7), gyratory rotation system (8) and liquid-overlay technique (9). While the established 3-D tumor models have been successfully used for the evaluation of the effects of drugs (10–12), they are not suitable for the screening of immune cells. Our previous study used a tissue engineering approach and type I collagen hydrogel as a matrix to successfully create hepatic tissue *in vitro*(13). However, whether the engineered 3-D tumor tissues cultured with a similar approach may be used for evaluating the efficacy of antitumor immune cells has not been explored. Given that immune and tumor cell interactions in the engineered 3-D tumor model resemble those *in vivo*, we hypothesized that the evaluation of the antitumor activity of immune cells in an engineered 3-D tumor model may be better than that in a 2-D tumor culture model.

In the present study, we first employed tissue engineering technology to establish an *in vitro* 3-D gastric tumor model. Furthermore, we compared the growth of tumor cells and the cytotoxicity of cytokine-induced killer (CIK) cells in 2-D culture and the engineered 3-D tumor culture models. In addition, we compared the cytotoxicity of CIK cells, dendritic cell-activated CIK (DC-CIK) cells and anti-CEA/CD3 bi-specific single-chain antibody-activated CIK cells (CIK-CEA/CD3-bscAb) in the engineered 3-D model. Our results indicate that this engineered 3-D gastric tumor model may be a simple and useful approach to screen antitumor immune cells *in vitro*.

## Materials and methods

### 

#### Cell culture

The human undifferentiated stomach adenocarcinoma cell line BGC823 was purchased from the Chinese Academy of Medical Sciences (Beijing, China). Tumor cells were cultured in Dulbecco’s modified Eagle’s medium (DMEM; Sigma, St. Louis, MO, USA) containing 10% fetal calf serum (FCS; Hyclone, Logan, UT, USA) as monolayer growth in 25-cm^2^ plastic tissue culture flasks at 37°C in 5% CO_2_ and 95% humidity in an incubator (HF212UV; Heal Force, Shanghai, China).

#### Establishment of different types of gastric tumor cell culture models in vitro

BGC823 cells (2×10^5^) were mixed with 500 *μ*l collagen type I (2 mg/ml) prepared from Sprague Dawley rat tails (14) in 2X DMEM (500 *μ*l) and adjusted to pH 7.2 using 0.1 M NaOH on ice. Subsequently, the mixture was added to 96-well plates (1×10^4^ cells/50 *μ*l/well) and incubated at 37°C in 5% CO_2_ for 10–15 min, followed by adding 50 *μ*l complete culture media into each well for the establishment of the 3-D engineered model of gastric tumors *in vitro*.

Traditional gastric tumor culture models were established and used as controls. Briefly, BGC823 cells (1×10^4^ cells/well) were cultured in 96-well plates and used as a monolayer tumor culture model. The same numbers of BGC823 cells were cultured in 96-well plates that had been coated with collagen type I (30 *μ*l/well) and used as the liquid-overlay tumor culture model.

Following culture for varying periods of time, the cells in the monolayer and liquid-overlay models were stained with hematoxylin and eosin (HE) and directly examined by the Lab-Tell^®^ II Chamber slide system (Nunc, Roskilde, Denmark) on an inverted phase contrast microscope (TE2000-U, Nikon, Tokyo, Japan). The cells in the engineered 3-D model were fixed and paraffin-embedded, followed by staining with HE and examinination under a microscope.

#### Immune cell isolation and culture

Heparinized peripheral blood samples (50 ml) were obtained from healthy donors and peripheral blood mononuclear cells (PBMCs) were isolated by density gradient Ficoll-Paque centrifugation (TBD, Tianjin, China). PBMCs at 1×10^7^ cells/ml were incubated in Cellix 901 serum-free medium (SIMA, Beijing, China) in tissue culture flasks for 3 h and the non-adherent cells were harvested, followed by gentle washing. The adherent cells were exposed to fresh Cellix 901 medium containing rh-IL-4 (1,000 U/ml) and GM-CSF (1,000 U/ml) every other day for seven days. Then the DC cells were loaded with BGC823 tumor cell lysate (20 *μ*g/ml) in the presence of 3 *μ*l/ml *Pseudomonas aeruginosa* preparation (Weikexi, Haikou, China) and cultured for 48 h. The cells were harvested, washed and stained with fluorescent-conjugated antibodies against CD80, CD83, CD86 and HLA-DR (Becton Dickinson, San Diego, CA, USA), followed flow cytometry analysis on a FACScan (Becton Dickinson).

#### Generation of CIK and DC-CIK cells

The harvested non-adherent cells were stimulated with 1,000 U/ml IFN-γ (SIMA) in Cellix 601 serum-free medium (SIMA) at 37°C for 24 h and activated by anti-CD3 (20 *μ*g/ml) and 1 *μ*g/ml anti-CD28 (Mabworks Biotech, Beijing, China) in the presence of 1,000 U/ml IL-2 for 4–5 days to induce CIK cells. Subsequently, one portion of the CIK cells was mixed with mature DCs at a ratio of 100:1 and cultured for 1–2 days to generate DC-CIK cells. The remaining CIK cells were continually cultured for up to 13–14 days following stimulation. The phenotypes of CIK and DC-CIK cells were characterized by flow cytometry analysis using antibodies against CD3, CD4, CD8 and CD56 (Becton Dickinson).

#### Generation of CIK-CEA/CD3-bscAb cells

The harvested CIK cells (1×10^7^ cells) were stimulated with 10 *μ*g anti-CEA/anti-CD3 bi-specific Ab (ABT, Beijing, China) at 4°C for 30 min and cultured in Cellix 601 serum-free medium.

#### Tumor cell transfection and CIK cell labeling

BGC823 cells (1×10^6^) were transfected with 6 *μ*g pEGFP-C1 plasmid (Clontech, Mountain View, CA, USA) using the X-fect transfection reagent (Clontech) according to the manufacturer’s instructions. The CIK cells (2×10^7^ cells) were labeled with 8 *μ*l PKH26 (Sigma), according to the manufacturer’s instructions. The efficiency of cell transfection and labeling was examined by flow cytometry analysis.

### Antitumor activity of CIK cells in different tumor growth models

#### Morphology

Unmanipulated and pEGFP-C1-transfected BGC823 cells were cultured in 96-well plates for 24 h for the generation of different gastric tumor growth models. Subsequently, individual wells of tumor cells were reacted in triplicate with CIK and PKH26-labeled CIK cells (1×10^5^ cells/well) at an effector:target cell (E:T) ratio of 10:1. The morphology of tumor cells was examined under an inverted phase contrast microscope, and a confocal laser scanning microscope (ACAS-208, Olympus, Tokyo, Japan) was used to observe the immune cell-tumor cell interactions and the antitumor activity of CIK cells.

#### Cytotoxic effects

The cytotoxicity of CIK cells against individual types of tumor cells was measured by lactate dehydrogenase (LDH) assay using the CytoTox 96 nonradioactive cytotoxicity assay kit (Promega, Madison, WI, USA), according to the manufacturer’s instructions. Briefly, BGC823 cells (1×10^4^/well) were cultured for 24 h and then reacted in triplicate with 5×10^4^ cells/well or 1×10^5^ cells/well CIK cells (E:T ratios of 5–10:1). The BGC823 cells were used as the target cells and the CIK cells as the effector cells. The BGC823 and CIK cells alone were used as controls (Target spontaneous and Effector spontaneous), and BGC823 cells treated with lysis solution were used as maximum control (Target maximum). The supernatants of the cultured cells were harvested, and the levels of LDH in the supernatants were determined by reading at 492 nm on an ELISA analyzer (DNM-9602G, Perlong, Beijing, China). The percentages of specific cytotoxicity were calculated by the equation: %Cytotoxicity = [A (Experimental) − A (Effector Spontaneous) − A (Target Spontaneous)] × 100 / [A (Target maximum) − A (Target spontaneous)].

Following co-culture of the engineered gastric tumor cells with CIK cell for varying periods of time, these cells were fixed and paraffin-embedded. Subsequently, the engineered gastric tumor sections were stained with HE and examined under a light microscope.

#### Cytotoxicity of different immune cells against the engineered 3-D model of tumor cells

Following the establishment of the engineered 3-D model in 96-well plates overnight, the tumor cells were reacted in triplicate with 100 *μ*l (1×10^5^ cells) CIK, DC-CIK and CIK-CEA/CD3-bscAb cells at an estimated E:T ratio of 10:1. The engineered 3-D tumor cells or each type of CIK cells alone were used as negative controls, while the 3-D tumor cells that had been treated with lysis solution were used as maximum controls. The supernatants were harvested and the cytotoxicity of different types of CIK cells against the tumor cells was determined by LDH assays.

#### Statistical analysis

Data are expressed as the mean ± SD. The difference between groups was analyzed by ANOVA and Student’s t-test using the SPSS 17.0 statistical software (SPSS, Inc., Chicago, IL, USA). P<0.05 was considered to indicate a statistically significant result.

## Results

### 

#### Cancer cells form spheroids in collagen matrix

**Figure 1.**
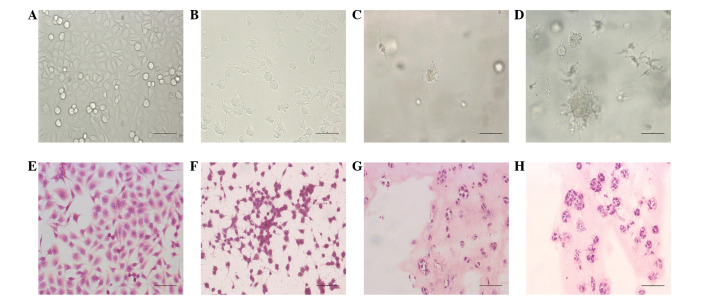
Morphological analysis of different types of tumor culture models *in vitro*. BGC823 cells (1×10^4^ cells/well) were cultured in 96-well plates and used as a monolayer tumor culture model. BGC823 cells were cultured in 96-well plates that had been coated with collagen type I (30 *μ*l/well) and used as the liquid-overlay tumor culture model. BGC823 cells were mixed with rat-tail collagen type I in 2X DMEM, pH 7.2, and cultured in complete culture media for the establishment of a 3-D engineered model of gastric tumors *in vitro*. Following culture for 24 or 96 h, the tumor cells were observed under a light microscope and stained with HE. Data shown are representative images (magnification, ×200) of the tumor cells in different types of models. (A) The monolayer tumor model at 24 h; (B) the liquid-overlay tumor culture model at 24 h; (C) the 3-D tumor culture model at 24 h; (D) the 3-D tumor culture model at 96 h (evident spheroids); (E) histological analysis of the monolayer tumor model at 24 h; (F) histological analysis of the liquid-overlay tumor culture model at 24 h; (G) histological analysis of the 3-D tumor culture model at 24 h; and (H) histological analysis of the 3-D tumor culture model at 96 h. Scale bar equals 100 *μ*m. HE, hematoxylin and eosin; DMEM, Dulbecco’s modified Eagle’s medium; 3-D, 3-dimensional.

Compared with other tumor culture methods (Fig. 1A and B), human gastric cancer (BGC823) cells grew in 3-D culture and formed tumor spheroids in the engineered culture model (Fig. 1C). Phase contrast micrograph images showed that the numbers and sizes of tumor cells spheroids increased with time (Fig. 1D). Histological characterization revealed that the tumor cells formed numerous spheroids in the collagen matrix in the engineered culture model (Fig. 1G and H). However, there were no tumor cell spheroids in the monolayer culture (Fig. 1E and F).

#### Characterization of immune cells

To study the interaction of immune cells with tumor cells in different culture models, we prepared human DCs and CIK and DC-CIK cells *in vitro*. Flow cytometry analysis indicated that the CIK cells were >95% CD3^+^ and 70% CD8^+^, but only ∼30% CD3^+^CD56^+^ cells. Similarly, the purity of DC-CIK cells was >90% and the majority of DC-CIK cells were CD3^+^, CD4^+^, CD8^+^ and CD56^+^. The generated mature DCs had 90% purity and the majority of DCs had high levels of CD80, CD86, CD83 and HLA-DR expression.

### CIK cells migrate into the matrix and kill cancer cells

**Figure 2.**
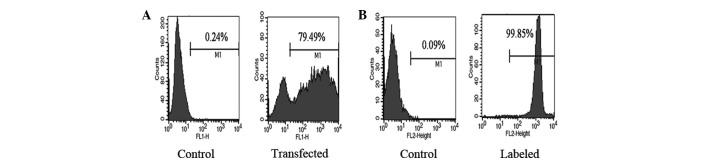
FACS analysis of the transfected tumor cells and the PKH26-labeled CIK cells. BGC823 cells were transfected with EGFP-expressing plasmid and the CIK cells were labeled with PKH26. The efficacy of transfection and PKH-labeling was characterized by FACS analysis. Data are representative histograms of each type of cells from three separate experiments. (A) FACS analysis of EGFP^+^ BGC823 cells; (B) FACS analysis of PKH26-labeled CIK cells. CIK, cytokine-induced killer.

**Figure 3.**
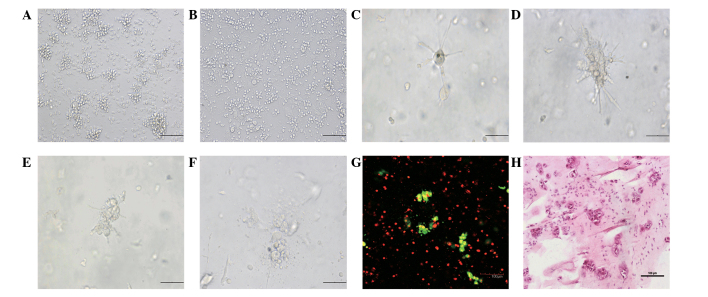
Cytotoxic process of CIK cells against tumor cells in different types of tumor culture models *in vitro*. The same numbers of CIK cells were added to the monolayer, liquid-overlay and 3-D gastric tumor cell culture models and the cytotoxic process of CIK cells against the tumor cells was characterized by longitudinal morphological observation, histological examination and confocal analysis. Data shown are representative images of different groups of cells from three separate experiments. (A) The cytotoxicity of CIK cells against the monolayer tumor cells at 12 h post-interaction; (B) the cytotoxicity of CIK cells against the liquid-overlay tumor cells at 12 h post-interaction; (C) the structure of intact tumor spheroids at 12 h post-CIK-tumor cell interaction; (D) the CIK cells migrated and surrounded the tumor spheroids at 24 h post-CIK-tumor cell interaction; (E) the CIK cells invaded the tumor spheroids at 36 h post-CIK-tumor cell interaction; (F) the CIK cells destroyed the tumor spheroids at 48 h post-CIK-tumor cell interaction; (G) the confocal laser scanning microscopy of the interaction of CIK cells with the tumor spheroids at 24 h post-CIK-tumor cell interaction; and (H) histological examination of the interaction of CIK cells with the tumor spheroids at 24 h post-CIK-tumor cell interaction. (A, B, G and H) Magnification, ×200; scale bar equals 100 *μ*m. (C, D, E and F) magnification, ×400; scale bar equals 50 *μ*m. CIK, cytokine-induced killer; 3-D, 3-dimensional.

To visualize the interaction of CIK cells with tumor cells, we transfected tumor cells with pEGFP-C1 plasmids and found that 79.49% of the tumor cells were GFP-positive (Fig. 2A). Similarly, we labeled the CIK cells with PKH26 and found that 99.85% of CIK cells exhibited a red color (Fig. 2B). After adding CIK cells to the different tumor culture models, we found that the CIK cells had direct contact with tumor cells in the control models within a short time (Fig. 3A and B). By contrast, the CIK cells first infiltrated into the collagen matrix (Fig. 3C) and then migrated towards and surrounded the tumor spheroids (Fig. 3D). Finally, the CIK cells destroyed the spheroids (Fig. 3E) and killed the tumor cells (Fig. 3F). Confocal laser scanning and histological analyses indicated that the CIK cells migrated into the collagen matrix and destroyed the spheroids and tumor cells (Fig. 3G and H).

#### Cytotoxicity of CIK cells in different tumor culture models

**Figure 4.**
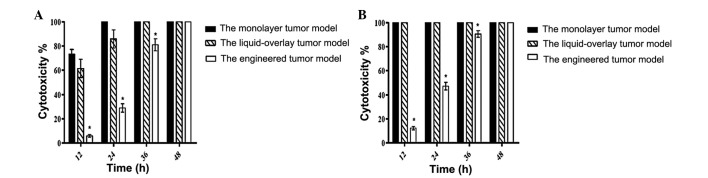
Cytotoxicity of CIK cells against the tumor cells in different tumor culture models. CIK cells were added to the monolayer, liquid-overlay and 3-D tumor culture models at E:T ratios of 5–10:1 for 12–36 h. The cytotoxicity of CIK cells against the tumor cells in these models was determined by LDH assays. The values are the mean percentage ± standard deviation (SD) of each group of cells from three separate experiments. (A) The cytotoxicity (%) at E:T ratio of 5:1; (B) the cytotoxicity (%) at E:T ratio of 10:1. ^*^P<0.05 vs. the other groups. CIK, cytokine-induced killer; 3-D, 3-dimensional; LDH, lactate dehydrogenase; E:T, effector:target cell.

Quantitative analysis of the cytotoxicity of CIK cells in different tumor culture models revealed that following interaction of CIK cells with tumor cells for varying periods of time, the cytotoxicity of CIK cells against tumor cells in the engineered tumor culture model at 12 h post-interaction was low and gradually increased over time. The cytotoxicity of CIK cells against tumor cells in the engineered tumor culture models at 12, 24 and 36 h post-interaction was significantly lower than that in the control models regardless of the ratios of E:T in our experimental system (Fig. 4). However, there was no significant difference in the cytotoxicity against tumor cells among the tested models at 48 h post-interaction.

#### Antitumor activity of different immune cells in the 3-D model

**Figure 5.**
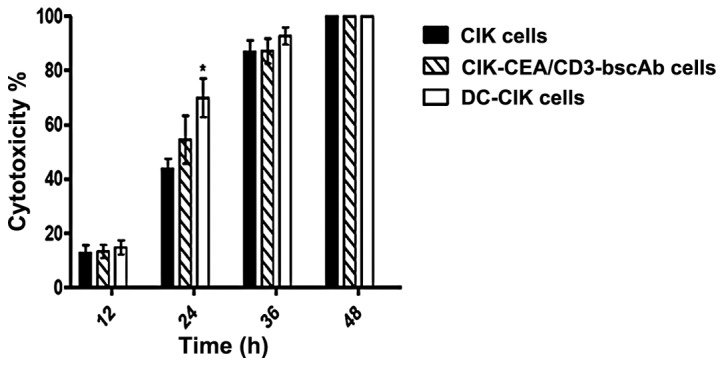
Cytotoxicity of different immune cells against the tumor cells in the engineered 3-D tumor culture model. CIK, CIK-CEA/CD3-bscAb and DC-CIK cells were added into the engineered 3-D tumor model at an E:T ratio of 10:1 and the cytotoxicity of different types of immune cells against the tumor cells was determined by LDH assays. The values shown are the mean percentage ± SD of the cytotoxicity of each type of immune cells from three separate experiments. ^*^P<0.05 vs. other groups. CIK, cytokine-induced killer; CIK-CEA/CD3-bscAb, anti-CEA/CD3 bi-specific single-chain antibody-activated CIK; DC-CIK, dendritic cell-activated CIK; LDH, lactate dehydrogenase; E:T, effector:target cell.

Finally, we employed the engineered tumor culture model to examine the cytotoxicity of CIK cells, DC-CIK cells and CIK-CEA/CD3-bscAb cells following interaction with tumor cells for varying periods. As shown in Fig. 5, there was no significant difference in the cytotoxicity of different types of CIK cells against tumor cells at 12 h post-interaction. Notably, the cytotoxicity of DC-CIK cells against tumor cells at 24 h was significantly stronger than that of CIK and CIK-CEA/CD3-bscAb cells. Following interaction with tumor cells for 36 and 48 h, the cytotoxicity of the three types of CIK cells against tumor cells in this model displayed similar levels.

## Discussion

The 2-D cell culture models have been widely used for studying the formation, function and pathology of tumors (15). It has been suggested that 2-D cell cultures do not accurately reflect the cellular environment *in vivo* due to the significant differences in the spatial array and cellular surroundings (16). In the present study, we used the tissue engineering approach to establish a 3-D gastric tumor culture model *in vitro*. Our results showed that tumor cells grew in the collagen matrix and formed spheroids in the matrix. The engineered tumor 3-D culture model has a 3-D structure and should be better than the 2-D cell culture models for mimicking the tumor growth *in vivo*.

Engineering tissues have been traditionally developed for the repair of damaged tissues and the reconstruction of organs. The engineered scaffold materials to build the 3-D tumor models have been used for evaluating the activity of antitumor drugs *in vitro*(17–19). There are few studies using the engineered tumor models to evaluate the antitumor activity of immune cells *in vitro*. In the present study, we used collagen as the scaffold material to establish a tumor 3-D culture model. Collagen is a commonly used scaffold material in tissue engineering and is also the primary structural protein of ECM that maintains the stability of the basement membrane (20). Collagen has a porous structure that supports tumor cell growth to form a 3-D tumor-like structure. The porous structure of the scaffold provides a huge surface for cells to adhere to and to interact with the scaffold. The porous structure also allows the migration or entry of cultured cells into the scaffold and nutrients to diffuse into the scaffold to support the growth of the seeded cells (21). In addition, while collagen is liquid at a low temperature, it is polymerized to form a collagen gel after increasing the temperature and adjusting the pH to alkaline. Moreover, the collagen gel is translucent and can be easily paraffin-embedded, which allows the examination of the immune and tumor cell interactions by microscopy. Therefore, collagen has a number of advantages in establishing the 3-D tumor model for evaluating the antitumor activity of immune cells *in vitro*.

Immunotherapies have been widely used for the treatment of cancer patients in the clinic (22–25). DCs are important antigen-presenting cells and T cells are critical for antitumor immunity. Currently, the antitumor activity of immune cells *in vitro* is primarily evaluated by the 2-D cell culture (26,27). In this experimental system, the immune cells are able to contact the tumor cells directly, leading to cytotoxicity against tumor cells. However, this model poorly reflects the cytotoxic process of immune cells against tumor cells *in vivo*, as tumor cells are surrounded by ECM *in vivo*. Indeed, immune cells have to migrate through the ECM barriers, such as the basement membrane (28). Collagen is the primary structural protein of ECM and may be considered as an artificial ECM (29). We found that tumor cells formed spheroids in the engineered 3-D tumor culture model and that the cytotoxic process of CIK cells against tumor cells in the 3-D tumor culture model was slower than that in the 2-D tumor cell culture models. We observed that CIK cells migrated in the collagen matrix, surrounded the tumor spheroids, invaded the tumor spheroids and finally killed the tumor cells. Therefore, the cytotoxic process of immune cells in the engineered 3-D tumor culture mode may be similar to that *in vivo*.

Antigen-specific T cells have strong cytotoxicity against tumor cells. CEA is expressed by BGC823 tumor cells, and the CEA/CD3 bi-specific single-chain antibody inhibits the growth of CEA^+^ tumor cells *in vivo*(30). We compared the cytotoxicity of CIK, DC-CIK and CIK-CEA/CD3-bscAb cells in the engineered 3-D tumor culture model. We found that DC-CIK cells displayed early and strong cytotoxicity against the tumor cells, although the cytotoxicity of these immune effector cells was comparable at 48 h post-interaction in the engineered 3-D tumor culture cell model. Therefore, the engineered 3-D tumor culture model may be used for evaluating the cytotoxicity of different types of immune cells *in vitro*.

In conclusion, we used collagen as the matrix to establish an engineered gastric tumor 3-D culture model, in which gastric tumor cells formed spheroids. Furthermore, our data indicated that the established engineered 3-D tumor culture model was used for effectively evaluating the cytotoxicity of different types of activated immune cells *in vitro*.
